# Prevalence of Intestinal Parasites among Immunocompromised Patients, Children, and Adults in Sana'a, Yemen

**DOI:** 10.1155/2022/5976640

**Published:** 2022-06-08

**Authors:** Asma Al-Yousofi, Yongmin Yan, Abdulsalam M. Al_Mekhlafi, Kamal Hezam, Fatma A. Abouelnazar, Balqees Al-Rateb, Hafsah Almamary, Rasheed Abdulwase

**Affiliations:** ^1^School of Medicine, Jiangsu University, Zhenjiang, Jiangsu, China; ^2^School of Medical Science and Laboratory Medicine, Jiangsu University, Zhenjiang, Jiangsu, China; ^3^Department of Parasitology, Faculty of Medicine and Health Sciences, Sana'a University, Sanaa, Yemen; ^4^School of Medicine, Nankai University, Nankai, China; ^5^Department of Medical Laboratory, Sana'a University, Sanaa, Yemen; ^6^School of Management, Jiangsu University, Zhenjiang, Jiangsu, China

## Abstract

Intestinal parasite infection (IPI) is still a very important public health issue. The severity of the parasitic disease has been reported as a high infection in immunocompromised patients and children. Hence, this study aimed to investigate the prevalence of intestinal parasites among immunocompromised patients and children with various gastrointestinal system complications in Sana'a city, Yemen, with different variables, including genus and age, and explore the risk factors associated with parasitic intestinal infections. The study socioeconomic data and certain behavioral and environmental risk factors and stool samples were collected from immunocompromised adult and children's patients, including children (one to eight years old), pregnant women, diabetes mellitus patients, cancer patients, HIV patients, and older adults. Out of 436 fecal samples, the overall prevalence rate of IPIs among immunocompromised patients and children in Sana'a was 51.8%. In contrast, the rate of infection in children (26.1%) was higher than that in old patients (25.7%) and in females (38.5%) and higher than that in males (13.3%). The protozoa (44.5%) have been shown more than intestinal helminths (7.3%) in samples, and the most common intestinal protozoan was *Giardia lamblia* and *Entamoeba histolytica* (13.8% and 12.8%), respectively. The most common intestinal helminthiasis was *Hymenolepis nana* with 1.8%. Concluding that the rate of infection was high for several reasons, including lack of commitment to hygiene as not handwashing after using the toilet (88.9%), eating uncovered food (56.3%), poor sanitation as lack of water sources (59.5%), reduced health education, and presence of other family members infected by parasites (61.3%). Interventions are required to reduce intestinal parasites, including health education on personal hygiene for patients, increasing awareness, and improving the environment and healthcare system.

## 1. Introduction

Intestinal parasitic infections are still a significant public health problem globally, especially in developing countries. Intestinal parasitic infections result from risk factors, such as environmental, socioeconomic, and behavioral conditions [1]. Parasitic infections in food handlers may be a real risk to those more prone to diseases like hospitalized patients, especially those who suffer from immune-deficient conditions [2]. In fact, there is a higher risk for opportunistic pathogen infections in immunocompromised individuals and children. The main intestinal parasites that infect humans are *Entamoeba histolytica*, *Balantidium coli*, *Giardia lamblia*, *Isospora belli*, *Cryptosporidium* species, Taenia solium, *Hymenolepis nana*, *Diphyllobothrium latum*, *Ascaris lumbricoides*, *Trichuris trichiura*, *Enterobius vermicularis*, and *Schistosoma mansoni* [3, 4]. There are also species whose pathogenicity is debatable, such as Blastocystis sp. And Dientamoeba fragilis. Because their presence is not associated with clinical significance, and their role in patients with gastrointestinal symptoms is currently controversial. However, most laboratories and clinicians do not routinely test for *Blastocystis* sp. and *D. fragilis* [5]. Enteric parasitic infections are spread among the widest of all chronic human infections worldwide [6]. The opportunistic infection happens in patients with weak host defenses caused by infectious agents that do not normally produce disease in healthy individuals. These are mutual in immunocompromised patients. Immunocompromised is a condition where a person's immune system is weak or absent [7].

Immunocompromised people and children are with less ability to fight infections due to the immune response that does not correctly function such as in children, pregnant women, patients who have HIV (human immunodeficiency virus), or the patients undergoing chemotherapy or radiation therapy for cancer [8]. Additional cases such as genetic disorders and certain cancers can also cause a person to become immunocompromised [9]. Immunocompromised patients can sometimes be susceptible to more severe infections and complications than healthy people. They are also more vulnerable to opportunistic infections, which generally do not afflict healthy individuals [10].

The parasite is the organism that gets water or food and shelter in or on another organism. Still, it does not give anything for the survival of the host; for example, the parasite in the body would live on the cells, the food. As a result, the parasite causes malnourishment, which we do not require to stay healthy and be active. Moreover, it weakens the human body and puts it at risk of illness, disease, and different health challenges [11, 12].

Parasitic infections continue to be a serious problem for immunocompromised people and children in resource-poor settings, and more effort is needed to develop accessible diagnostic tests as well as to understand better and control their pathogenic effects. New ideas regarding how intestinal parasites interact with host immunity point to the need for more epidemiological and clinical data to fully understand the intricacies of such immunological interactions [13].

Yemen is a developing Middle Eastern country. It depends completely on groundwater and rainwater as a source of water. In recent times, Yemen has fallen by a deepwater crisis characterized through a very rapid withdrawal of groundwater, limited access of the population to safe drinking water, and extreme water supply lacks in the major cities [14]. The World Health Organization in 2009 has reported that only 25% of the population had easy access to safe water [15]. The current water shortage has stimulated a high incidence of infections of the intestinal parasite in Yemen [16].

The infection of the intestinal parasite is highly predominant in Yemen, especially in rural areas. These infections accompany social status and geographical environment and are highly distributed among children because they are swimming in contaminated water with freshwater snails and are playing in the soil that may contain eggs of parasites that grow in soil [17]. These children were suffering from symptoms such as abdominal pain, diarrhea, nausea, vomiting, and sometimes itching in the anal area. The parasitic intestinal infection may cause public health problems, such as mental health problems and iron deficiency anemia [18]. We research this parasite in the Sana'a city area and study risk factors and introduce recommendations that may help to reduce the frequent infection in the study area.

Studies conducted in Sana'a were mostly conducted in healthy people; the situation of IPIs in immunocompromised patients and children of Sana'a remains neglected to a great extent. Therefore, the present study was designed to determine the prevalence of intestinal parasites and associated risk factors among immunocompromised patients and children of Sana'a, north of Yemen.

## 2. Methodology

### 2.1. Study Design

This investigation was a cross-sectional study conducted in Sana'a city between December 2017 and December 2018. The structured questionnaire was utilized to collect relevant clinical data of the participants and sociodemographic characteristics. The study's protocol was approved by the Research Ethics Committee of the Faculty of Medicine and Health Sciences at Sana'a University in Yemen.

### 2.2. Study Area and Study Population

**Figure 1:**
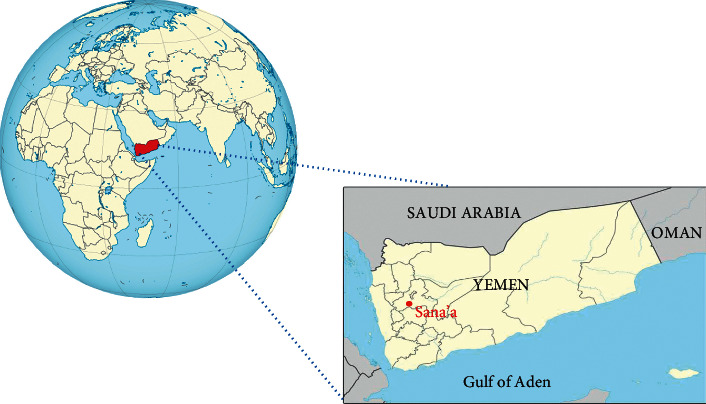
Map of Yemen showing Sana'a city.

The study was carried out by people in Sana'a city (Figure 1) [19]. The tool samples were collected from immunocompromised patients and children (children from 1 year to eight years of age, pregnant women, diabetes mellitus patients, cancer patients, HIV patients, and older adults (more than 64 years old) to analyze for the intestinal parasite) who attended to hospital. The fecal samples were collected from people referred to the parasitological lab for fecal examination. The study population was selected randomly from urban governmental hospitals. Epidemiologic and socioeconomic data were collected using a pretested structured questionnaire, covering the important, relevant aspects of this descriptive, analytical, and cross-sectional study.

### 2.3. Sample Size

The total samples were 436 collected from participants who were invited to participate voluntarily after the precise explanation of the study's objectives and gain informed consent from the hospital and their guardians before collecting data and samples. Parents wrote the information if the participants were children. Samples were examined and processed for protozoa and helminth. The parasitological data were collected through analyzing stool samples and using formalin ethyl-acetate concentration technique.

### 2.4. Sample Collection and Analysis

Data from each participant were collected by using a predesigned questionnaire. A single fresh stool sample collected from each participant into sterile and labeled containers and analyzed for 24 hours of the collection were transported immediately to the Parasitology Laboratory of the Faculty of Medicine and Health Sciences, Sana'a University. The stool samples were initially examined macroscopically to evaluate stool color, consistency, and content as the presence of blood, mucus, and segments of worms. The stool samples were tested in iodine and saline wet preparation and using direct analysis. As soon as possible after collection, perform a microscopic inspection with 10× and 40× objectives. In addition, after concentration, stool sediments were analyzed using the formol-ether sedimentation technique according to normal guidelines [20]. The remaining portion of the specimen was stored in 10% formalin for the concentration technique. The positive results have been recorded as segments of worms, eggs, cysts, and trophozoites.

### 2.5. Data Analysis

SPSS software (version 26) and Graph Pad Prism (version7.00) analyzed the study's data. The data such as age and gender of the participants, type of intestinal protozoa, and helminth with the number of participants were presented as numbers and percentages. The IBM SPSS Statistics version 26.0 for Windows (IBM Corp., Armonk, NY, USA) was used in the data analysis. Categorical variables were reported as frequencies and percentages. Chi-square or Fisher's exact tests were used to test the significance of differences or associations between the variables, which were considered statistically significant at *P* values <60.05. Furthermore, univariate and multivariable logistic regression approaches were employed to identify potential risk factors associated with IPIs.

## 3. Results

### 3.1. Prevalence of Parasitic Intestinal Infections by Species

**Figure 2:**
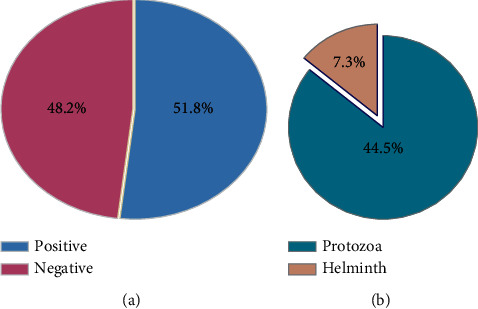
(a) Infected and noninfected participants. (b) The prevalence of intestinal parasites.

Out of 436 participants were recruited in the present study, 222 were children, and 214 were old patients. Of them, 252 were females, and 184 were males. The overall prevalence rate of IPIs among the 436 examined immunocompromised patients and children in Sana'a was 226 (51.8%), and 210 (48.2%) were negative by intestinal parasites. The most prevalent type of intestinal parasites is protozoa, compared with the intestinal helminths being 44.5% and 7.3%, respectively, as shown in Figures 2(a) and 2(b).

**Figure 3:**
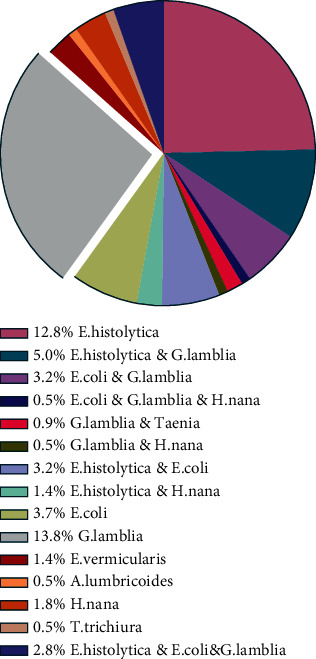
Comparison of the prevalence (%) of intestinal parasitic species among participants.

**Figure 4:**
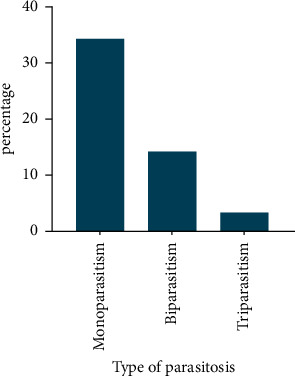
Distribution of the type of parasitism in the sample studied.

The most common intestinal protozoa are *G*. *lamblia* (13.8%) and *E*. *histolytica* (12.8%). On the other hand, the most common intestinal helminths are *H. nana* (1.8%), followed by *E*. *vermicularis* (1.4%). However, *A*. *lumbricoides* and *T*. *trichiura* were the least frequent helminths among participants.  Figure 3 shows the interpretation. Regarding the multiplicity of infections, the majority of participants (34.3%) were infected with a single parasite species. However, 14.2% and 3.3% of cases had double and triple infections, respectively, as shown in Figure 4.

### 3.2. Distribution of Intestinal Parasitic Infections by Gender

**Figure 5:**
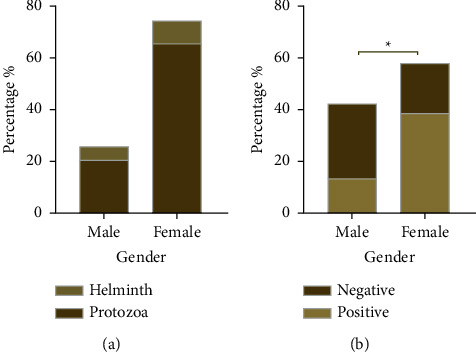
Parasitic infection rate according to gender. *Note*. ^*∗*^Significant at level *P* < 0.05.

Table 1 and Figure 5(a) show a statistically significant difference between the overall infection according to the gender, where the prevalence of infection was significantly higher (*P* ≤ 0.001) among females (38.5%) than males (13.3%, in addition, infection rates of helminth and protozoa according to the gender in females (8.8 vs. 65.5%, resp.) and (5.3 vs. 20.4%, resp.) in males, as presented in Figure 4(b). Both males and females showed comparable infection rates with *G*. *lamblia* (4.1% vs. 9.6%, resp.) and *E*. *histolytica* (2.8% vs. 10.1%, resp.). Notably, female participants showed significantly higher infection rates with *E*. *histolytica* than males (*P*=0.00249). Both males and females showed comparable infection rates with *Hymenolepis nana* (0.0 vs. 1.8%, resp.) and *Enterobius vermicularis* (0.5 vs. 0.9%, resp.) with no statistically significant differences.

### 3.3. Distribution of Intestinal Parasitic Infections by Age

**Figure 6:**
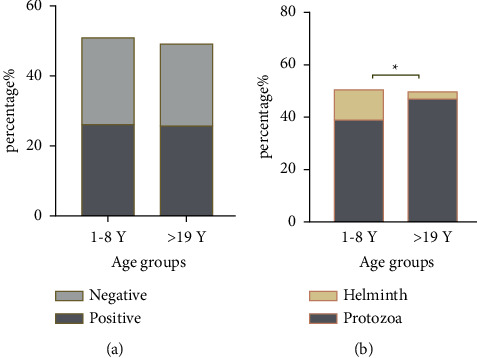
(a, b) Parasitic infection rate according to the age of participants. *Note*. ^*∗*^Significant at level *P* < 0.05.

Figure 6(a) shows comparable overall infection rates among participants ≤8 and 19 years old. A higher proportion was found in children with age less than eight years (26.1%) more than in older participants (25.7%), with no significant difference in the prevalence and distribution of infection. The difference between protozoa and helminths was statistically significant (*P* < 0.0132), as shown in Figure 6(b). However, participants ≤8 years and >19 years old were found for both helminths (11.5% vs. 2.7%, resp.) and protozoa (38.9 vs. 46.9%, resp.), with no statistically significant differences by species, as shown in Table 2.

### 3.4. Risk Factors Associated with Intestinal Parasitic Infections

We have found the rate of infection was high for several reasons; regarding the socioeconomic characteristics, univariate analysis showed that gender (OR = 4.345% CI: 2.893–6.525, *P* ≤ 0.001), type of house (OR = 1.877% CI: 1.28–2.74, *P* ≤ 0.001), source of water (OR = 2.090% CI: 1.42–3.076, *P* ≤ 0.001), and number of rooms (OR = 0.580% CI: 0.396–0.849, *P*=0.005) were significantly associated with IPIs among participants. However, the age group, family member size, occupation status, and literacy status were not significantly associated with IPIs among the participants. Regarding the behaviors and knowledge possibly associated with IPIs among participants, eating uncovered food (OR = 2.169% CI: 1.050–2.251, *P*=0.027), not washing hands after using toilets (OR = 7.924% CI: 1.799–34.89, *P*=0.001), not wearing shoes in toilet (OR = 4.457% CI: 1.483–13.394, *P*=0.004), not clipping nails weekly (OR = 2.333% CI: 1.154–4.719, *P*=0.016), and eating restaurant food (OR = 2.169% CI: 1.375–3.421, *P*=0.001) were significantly associated with IPIs among participants. However, showering times and swimming in stagnant water were not significantly associated with IPIs. And from the other side, regarding the environmental factors, the presence of animal risk factors is significantly associated with IPIs among participants (OR = 2.536% CI: 1.724–3.730, *P* ≤ 0.001). According to the symptoms that the participants were suffering from, multivariate analysis showed that suffering from itching (OR = 5.115% CI: 3.207–8.157, *P* ≤ 0.001), diarrhea (OR = 2.704% CI: 1.678–4.307, *P* ≤ 0.001), and the presence of other family members infected by parasites (OR = 1.799% CI: 1.203–2.691, *P*=0.004) were significantly associated with IPIs among participants. However, persistent abdominal pain was not significantly associated with IPIs (Table 3).

## 4. Discussion

The parasitic intestinal infections in many communities are still public health problems, mostly between children in the rural areas of developing countries [21]. Giardiasis is the most common intestinal protozoan infection globally [4]. *A*. *lumbricoides* is the second most common intestinal parasite in the world [22]. The most endemic areas worldwide are Asiatic countries, such as India, and the other developing countries [23]. Intestinal protozoan infections cause greater morbidity and mortality in the immunocompromised host [24].

Our study revealed a high prevalence rate of IPIs (51.8%) among immunocompromised patients and children. In the same context, the overall prevalence of IPIs among schoolchildren in the rural communities of Sana'a, Yemen, was 54.8% [25]. In addition, it is also consistent with that (57.0%) reported among schoolchildren in Ibb city [26], and in 3 Yemeni orphanages, the overall parasitic rate was 62.7% [27]. Further, our finding was higher than the study conducted in Taiz city; the prevalence of intestinal parasites was 27.8% [28]. In contrast, a higher prevalence rate of 90.0% has been reported for IPIs among primary school children in Al-Mahweet, northwest of Sana'a [29]. The other close study outside of Yemen in Riyadh, Saudi Arabia, was conducted to determine the prevalence of intestinal parasites among immunocompromised patients and children who were 39.7% positive for parasitic intestinal infections [30]. Also, a study conducted in Ethiopia showed the overall prevalence of intestinal parasitic infection (65.5%) [31].

These variances could be related to differences in the study population's features, geographical dispersion of sampling, diagnostic procedures, respondents' socioeconomic profile, sample size, sampling type, and sanitary conditions.

In our study, the predominance of protozoan infection was compared to helminthic infection (44.5 vs. 7.3%, resp.), where they were infected with one or more intestinal parasites. It was close to the findings of other studies among rural schoolchildren in Sana'a, with a predominance of protozoal compared to helminthic infections (37.6 vs. 17.2%) [25]. In addition, a similar result (40.3%) was reported by the study Prevalence of Intestinal Protozoan Infections among Patients conducted in Sana'a City Yemeni population [16]. The present infection finding was high compared to a previous study in Sana'a city, which was 30.9% [16]. In Saudi Arabia, among patients seeking health care was (27.8%–32.2%) [32], Iran (19.9%) [33], and Oman (18%) [34]. However, our study was lower compared to the prevalence of IPIs in Pakistan (52%) [35]; in Riyadh, Saudi Arabia, parasitic infection was related to protozoa in (95.6%) and intestinal worms in (4.4%) [36]. Parasitic infection in Iran was 95.33 and 4.87%, respectively, by helminths and protozoa [37].

The most common parasite detected was *Giardia lamblia* (13.8%) of isolated parasites. Inconsistent with other studies, Giardia duodenalis had the highest infection rate (17.7%), followed by *Entamoeba histolytica*/*dispar* (17.1%) [16]. In addition, in other studies, the highest IPIs among children were from Hadhramout; the main infective parasites were *Giardia lamblia* (19.2%) [38], and the second infection was in Ibb (23.6%) [39], followed by Saber camp, Lahaj governorate, Yemen (19.7%) [40]. In contrast, a lower *G. lamblia* infection rate of 3.0% was reported among children 3.0% from Al-Mahweet [29]. In the same vein, similar findings were found outside of Yemen, among the preschool children in Riyadh, Saudi Arabia. The most common parasites were *Giardia lamblia* (37.8%) [36]. Additionally, study of opportunistic parasitic infections among immunocompromised patients and children in Egypt showed the prevalence of *Giardia lamblia* (10%) [41]. Several studies in the Middle East have indicated that *Giardia lamblia* was the most common protozoan in Iran (53.9%, 10.78%, 8.0%, and 4.1%) [37, 42–44].

The prevalence of intestinal parasites differed greatly between the direct wet mount and formol-ether sedimentation techniques. The prevalence of intestinal parasites detected in direct wet mount and formol-ether sedimentation techniques could be attributed to differences in the characteristics of intestinal parasites; more trophozoite stages of protozoan parasite were detected in direct wet mount but not in formol-ether sedimentation technique.

The results showed that the study's second prevalent intestinal parasite was *E*. *histolytica* (12.8%). Another result was found among apparently healthy workers at restaurants of Sana'a city, where *E*. *histolytica* (48.87%) was the second prevalent intestinal parasite [45]. In addition, the infection rate of *E*. *histolytica* was 3.66 in the north of Baghdad [46].

This study revealed low prevalence rates of *H. nana* (1.8%), *E*. *vermicularis* (1.4%), and (0.5%) for both *A. lumbricoides* and *T*. *trichiura* were the lowest proportion of isolated parasites among immunocompromised patients and children; the same results were low prevalence rates of *H. nana* and *E*. *vermicularis* found among rural schoolchildren in Sana'a, being 5.3% and <0.5%, respectively [25]. The same result was conducted among apparently healthy workers at restaurants of Sana'a City, Yemen, which revealed *T*. *trichiura* (4.98%), *A. lumbricoides* (4.07%), *H. nana* (3.62%), and (0.91%) *E*. *vermicularis* [45]. Also, *A*. *lumbricoides* (0.4) and *H. nana* (2.4) were among schoolchildren of Sahar district, Yemen [47]. In contrast, another study revealed a high prevalence rate of *A. lumbricoides* (68%), *T*. *trichiura* (10%), and *H. nana* (13%) and a low prevalence rate of *E*. *vermicularis* (1%) among children in Ibb, Yemen [48]. In other countries such as Iran, *E*. *vermicularis* and *Hymenolepis nana* (0.2% and 0.9%), respectively [43]. Moreover, among food handlers of Swat, Khyber Pakhtunkhwa, Pakistan, the infection rate of *Ascaris lumbricoides* was 55.8%; Trichuris trichiura, 14.9%; *Enterobius vermicularis*, 9.73%; *Hymenolepis nana*, 9.36% [49].

Possibly the discrepancy might be attributable to the study subjects' geographical location, living conditions, and socioeconomic status. The low prevalence of *E*. *vermicularis* in such a vulnerable group is surprising; yet this parasite was identified through direct examination. The anal swab was taken with Scotch tape, which greatly improves detection [50]. Because local communities refused to use it, it was not used in this study. However, achieving good diagnostic sensitivity for all species is difficult. Traditional approaches like FEC (formol-ether concentration) are effective for detecting some intestinal helminth species, but they are insensitive to other parasite species. Although PCR can more accurately detect intestinal parasites, it is often not feasible in resource-poor situations, at least not in peripheral labs. Consequently, a more field-friendly, sensitive approach to on-the-spot intestinal parasite infection identification is required [51]. For example, in direct microscopic examination, *Entamoeba histolytica* and *Entamoeba dispar* are morphologically indistinguishable. As a result, specific antigen testing in the stool and a microscopic inspection are the preferred procedures for a reliable diagnosis of *E*. *histolytica* [52]. Additionally, because of less sensitive diagnostic procedures, infection with *Strongyloides stercoralis* and hookworm parasites is frequently underreported. The most sensitive approach for parasites in the larval stage is agar plate culture (APC) [53].

The multiple infections of the parasite are a common occurrence in this surveillance, where several types of intestinal parasites are encountered. Monoparasitism was detected in (34.3%) of patients, while polyparasitism was found in 17.2%, while biparasitism was found in 14.2%, and triparasitism in 3.3% of patients. Similarly, the parasitic infections among displaced persons in Yemen recently reported (36.9%) were infected by single parasite, whereas 7.4% were infected by two parasites and 1 case (0.3%) had three types of IPIs [40] in the same line. Single infections were the most dominant types of infections among school children, being prevalent among 34.6% of schoolchildren, while 8.5% double and 1.0% triple infections in rural communities of Sana'a, Yemen [25] Furthermore, the same results indicated that 54.1% of the positive cases were infected with one parasite, whereas 31.8%, 10.1%, and 4.1% were infected with two, three, and four parasites, respectively, among school children in Ibb, Yemen [26]. On the opposite finding, 14.5% of children had single infection and 75.5% had of multi-infection in Al-Mahweet, Yemen [29]. The previous study in outside of Yemen detected mixed parasites in 11.1% of infected patients, while single species parasitic infections were detected in 28.6% patients in Riyadh, Saudi Arabia [30]. Additionally, mixed infections were observed with five patients in immunocompromised child patients at a hospital in Izmir, Turkey [54]. In Egypt, mixed infection among immunocompromised patients was detected in 2 cases [41]. Moreover, Morocco has also found a higher prevalence of monoparasitism infection in 49 cases and polyparasitism infection in 20 cases of a positive sample [55]. Furthermore, mixed infections that were found among HIV-positive subjects in Hawassa Referral Hospital were 26.2% one, 21.5% two, 9.3% three, 2.3% four, and 0.5% five infections in Ethiopia [56–58].

This study revealed a higher prevalence of infection in females (38.5%) compared to males (13.3%). This result is statically significant; it agrees with the previous study that the infection rate among girls 31.5% was greater than boys 24.6% among children presenting to the Pediatric Centre in Sana'a, Yemen [59]. Furthermore, the females showed a higher prevalence of intestinal parasites infection (41.3%) than the males (26.4%). This is statistically significant (*P* < 0.05) in Morocco [55]. In Tehran primary school students, the infection rate was significantly lower among males than the females (17.4% versus 19.3%) [60] (10.62%) in males and (11.02%) in females, which shows statistical significance in Iran [37]. However, females were found to have a higher percentage of infection (59.42%) than the male group (29.50%). The association between gender and intestinal parasitic infection was statistically significant in Northwestern Saudi Arabia [61]. The prevalence of intestinal parasitic infections was slightly higher in females (0.58%) than males (0.38%) in the Riyadh region, Saudi Arabia [62]. Also, infection rates were higher among females than males (49.2% and 42.1%) among foreign workers in Madinah [63]. The same was found in Jeddah city, where women (48.7%) were more affected than men (47.8%) [64]. In Ethiopia, a study revealed the prevalence of *G*. *lamblia* infection was significantly different between males (21.3%) and females (32.1%); females were more infected than males [65]. On the contrary, in Al-Mahweet, Yemen, the infection rates were significantly higher among the males (46.5%) than in the females (43.5%) [29]. In addition, boys appeared to have a higher prevalence rate of giardiasis (32.1%) than girls (17.1%), and the difference was highly significant in Ibb, Yemen [26]. Another study in Riyadh, Saudi Arabia, was conducted among immunocompromised patients, which detected no significant difference in the rate of infection between female (38.8%) and male (39.3) patients [30]. In Egypt, males were affected more than females (20% versus 10%) among immunocompromised patients [41]. Moreover, in Egypt, opportunistic parasites were higher in males than in females (32.6% versus 12.9%) [66]. Similarly, infections in males (73.9%) were higher than in females (62.6%) among immunocompromised patients in Saudi Arabia [67].

The higher infection in females can be justified by considering that females are more exposed to the infective stages of parasitic infection due to the nature of the chores they perform in the house and their lifestyle. Females, on average, have more soil contact when cultivating vegetables and eat a raw vegetable with prepared food more frequently than males, and IPI was found to be relatively common among pregnant women. Our findings could be explained by the greater risk of females being exposed to contaminated waters because they are typically responsible for obtaining water and house working for the family in Yemen.

On the other hand, our study showed the prevalence of infection among participants is a higher proportion in children with age less than eight years (26.1%) more than in older participants (25.7%); a similar result in agreement with our result was in Lahaj, Yemen, which reported the highest prevalence in the age group 1–9 years as 45.5% [40]. Another study in Sana'a revealed that children ≤12 years (34%) had a higher prevalence than older patients >12 years (29.6%) [16]. Additionally, in Taiz city, the higher prevalence of infection in children ≤12 years 75.4% was more than in older patients >12 years 24.6% [68]. Another study documented that young children 5–17 years (71.4%) are more prone to infection than other age groups ≥18 years (28.6%) in Yemen [69]. This finding of our study is in accordance with the study finding outside of Yemen among indigenous communities in rural Malaysia where children under 13 years old (14.2%) had higher proportion when compared to adults over 12 years old (5.5%) [70]. In Indonesia, the rate of parasitic intestinal infection in immunocompromised children was 57% [71]. In Libya, the rate of infection was higher in children of less than ten years compared with children of 10 to 20 years or more than 20 years of age [72]. The prevalence of infection was higher among children aged <10 years compared to those aged ≥10 years (27.4% vs. 16.0%, resp.) in Malaysia [73]. The infection tends to be more common in children of 2–10 years old among immunocompromised patients in Riyadh, Saudi Arabia [30].

Children are a high-risk age group for intestinal parasite infection, and this group needs specific attention. These could be linked to the participants' frequent contact in overcrowded schools and health care centers, the sort of dirt and water they play with, the items they share, and a poor personal hygiene system that could favor parasite prevalence.

Intestinal parasite infections are commonly linked to living situations, poverty levels, personal and environmental hygiene, access to safe drinking water, health facility adequacy, and sanitation practices. Several studies showed that sociodemographic characteristics and associated factors contribute greatly to contract intestinal parasitic infections [74–76]. The effect of socioeconomic status on the risk of infectious diseases in general, and parasitic infections in particular, is complex and could be attributed to several other factors, such as lack of access to clean water, poor hygienic environment, and lack of access to education due to financial constraints and overcrowded conditions [77, 78]. This study showed that being female in gender was significantly associated with intestinal parasitic infections. The possible reason might be the involvement of females in house activities more than males. This could indicate that gender may or may not play a vital role in parasitosis that depends on the region and other environmental or behavioral factors [29]. After adjustment, the infection did not associate significantly with family occupation status; the same finding was discovered in Tehran primary school students [60]. Similar to studies in Thailand and Malaysia, there were no statistical differences in mothers' employment status between infected and uninfected individuals [79, 80].

In the present study, the type of house and number of rooms in participants' houses were significantly associated with intestinal parasitic maybe overcrowded of family members' conditions and a higher frequency of parasitic infections. A similar finding was shown in Tehran primary school students [60].

There are still some unhealthy habits among participants, mainly, eating uncovered food and entering the bathroom barefoot. This finding is in accordance with previous studies on Yemen and elsewhere [38, 45, 60, 81–84]. It is documented that handwashing is the essential hygienic practice for controlling pathogen transmission, including bacterial, viral, fungal, and parasitic agents [85–87]. In this regard, the current study revealed that not handwashing after using the toilet was a risk factor associated with acquiring IPIs, and this finding is consistent with a recent study in Taiz and Amran cities [68, 88].

This study found the history of symptoms to be positively associated with IPIs. Like many other diseases, abdominal pain in this study was a statistically significant factor in intestinal parasitic infections. The possible reason might be due to abdominal cramps, bloating, nausea and watery diarrhea during parasitic infections. Most intestinal parasitic infections were associated with diarrhea. In this study, having diarrhea also was significantly associated with parasitic infections. The possible reason might be due to the diarrhea-causing nature of intestinal parasites [89]. A similar result was found among Yemeni patients with cancer where diarrhea was associated with a higher risk of cryptosporidiosis and giardiasis in Sana'a, Yemen [90].

In the current study, other family members infected with IPIs are the significant infection risk factor. Previous studies reported a similar finding among children in Malaysia [21, 91]. This may indicate a high level of transmission of infection occurring horizontally within the household and infected family members serving as a source of infection. In this situation, parasites are probably transmitted directly through person-to-person contact.

## 5. Conclusion

This current study was indicated that immunocompromised people and children are more susceptible to parasitic intestinal infection. In contrast, intestinal protozoan infection is still a public health problem in Yemen, with the most common *G*. *lamblia* and *E*. *histolytica* infections. Statistical analysis indicated that low health education and inadequate personal hygiene were essential predictors for parasitic intestinal infections. As this study has been effectively highlighted to reduce this infection, a multisector effort is needed. Preventive measures should include good health practices and increased educational health programs on personal hygiene for patients and health services in all provinces, increasing awareness, and improving the environment and healthcare system, including rural areas. Moreover, it is also essential to find radical solutions to the recent water crises in Yemen.

## Figures and Tables

**Table 1:** Summarizing the distribution of parasitic species among participants according to gender.

Parasite species	Gender		*P* value
	Male *n* (%)	Female *n* (%)	
*Entamoeba histolytica*	12 (2.80)	44 (10.10)	0.00249^*∗*^
*Entamoeba coli*	6 (1.40)	10 (2.30)	1^a^
*Giardia lamblia*	18 (4.10)	42 (9.60)	0.167 ^a^
*Enterobius vermicularis*	2 (0.50)	4 (0.90)	1^a^
*Ascaris lumbricoides*	0 (0.00)	2 (0.50)	1^a^
*Hymenolepis nana*	0 (0.00)	8 (1.80)	0.1399^a^
*Trichuris trichiura*	2 (0.50)	0 (0.00)	0.422^a^

^a^Calculated for Fisher's exact test. *Note.*^*∗*^Significant at level *P* < 0.05.

**Table 2:** Summarizing the distribution of parasitic species among participants according to age.

Parasite species	Age		*P* value
	1–8 *n* (%)	>19 *n* (%)	
*Entamoeba histolytica*	24 (5.50)	16 (7.30)	0.4497
*Entamoeba coli*	8 (1.80)	4 (1.80)	1^a^
*Giardia lamblia*	36 (8.30)	12 (5.50)	0.273
*Enterobius vermicularis*	4 (0.90)	1 (0.50)	1^a^
*Ascaris lumbricoides*	2 (0.50)	0 (0.00)	1^a^
*Hymenolepis nana*	6 (1.40)	1 (0.50)	0.6218^a^
*Trichuris trichiura*	2 (0.50)	0 (0.00)	1^a^

^a^Calculated for Fisher's exact test.

**Table 3:** The prevalence of intestinal parasites according to the socioeconomic data, environmental factors, symptoms, behaviors, and epidemiological factors observed.

Variable	*N*	Positive *n* (%)	OR	95% CI	*P* value
*Gender*					
Male	184	58 (31.5)	4.345	2.893–6.525	0.001^*∗*^
Female	252	168 (66.7)			
*Age*					
1–8	222	114 (51.4)	10.04	0.714–1.515	0.837
>19	214	112 (52.3)			
*Literacy status of participants*					
Educated	200	102 (51.0)	0.686	0.318–1.482	0.082
Noneducated	72	30 (41.7)			
*Level of life*					
Good	414	214 (51.7)	1.121	0.474–2.653	0.794
Bad	22	12 (54.5)			
*Occupation status*					
Job	376	194 (51.6)	1.072	0.621–1.851	0.802
Jobless	60	32 (53.3)			
*Family member size*					
1–6	410	214 (52.2)	0.785	0.355–1.738	0.550
>6	26	12 (46.2)			
*Type of house*					
Own	218	96 (44)	1.877	1.28–2.74	0.001^*∗*^
Paying	218	130 (59.6)			
*Source of water*					
Public service	184	76 (41.3)	2.090	1.42–3.076	0.001^*∗*^
Car service of water	252	150 (59.5)			
*Number of rooms*					
1–3	242	140 (57.9)	0.580	0.396-.849	0.005^*∗*^
>3	194	86 (44.3)			
*Literacy status of breadwinner*					
Educated	408	216 (52.9)	0.494	0.223–1.0961	0.078
Noneducated	28	10 (35.7)			
*Presence of animal*					
No	210	84 (40.0)	2.536	1.724–3.730	0.001^*∗*^
Yes	226	142 (62.8)			
*Uncovered food*					
No	102	38 (37.3)	1.537	1.050–2.251	0.027^*∗*^
Yes	334	188 (56.3)			
*Handwashing after using toilets*					
Yes	418	210 (50.2)	7.924	1.799–34.89	0.001^*∗*^
No	18	16 (88.9)			
*Clipping fingernails*					
Yes	396	198 (50.0)	2.333	1.154 –4.719	0.016^∗^
No	40	28 (70.0)			
*Wearing shoes in the toilet*					
Yes	414	208 (50.2)	4.457	1.483–13.394	0.004^∗^
No	22	18 (81.8)			
*Swimming in stagnant water*					
No	408	212 (52.0)	0.925	0.430–1.988	0.841
Yes	28	14 (50.0)			
*Number of showering times/week*					
0-1	82	48 (58.5)	0.716	0.441–1.165	0.178
>1	354	178 (50.3)			
*Restaurant food*					
Yes	102	134 (52.0)	2.169	1.375–3.421	0.001^*∗*^
No	334	14 (50.0)			
*Bloody stool*					
No	386	38 (51.8)	1.007	0.559–1.817	0.980
Yes	50	188 (52.0)			
*Persistent abdominal pain*					
No	116	52 (44.8)	1.467	0.957–2.248	0.078
Yes	320	174 (54.3)			
*Itching*					
No	302	122 (40.4)	5.115	3.207–8.157	0.001^*∗*^
Yes	134	104 (77.6)			
*Diarrhea*					
No	102	34 (33.3)	2.704	1.678–4.307	0.001^*∗*^
Yes	334	192 (57.5)			
*Presence of other family members infected by parasites*					
No	286	134 (46.9)	1.799	1.203–2.691	0.004^*∗*^
Yes	150	92 (61.3)			

^*∗*^Significant key risk factors (*P* < 0.05). OR = odds ratio; CI = confidence interval. Reference group marked as OR = 1.0.

## Data Availability

Data are available from the corresponding author upon request.
